# Uniform Microscale Thermoresponsive Liposomes for Controlled Release Above Body Temperature

**DOI:** 10.1002/smll.74496

**Published:** 2026-07-11

**Authors:** S. Morteza Mousavi, Yang Zhang, Elvis Pandzic, David W. Inglis, Megan S. Lord, Robert A. Taylor, Ming Li

**Affiliations:** ^1^ School of Mechanical and Manufacturing Engineering University of New South Wales Sydney New South Wales Australia; ^2^ School of Engineering Macquarie University Sydney New South Wales Australia; ^3^ Katharina Gaus Light Microscopy Facility Mark Wainwright Analytical Centre University of New South Wales Sydney New South Wales Australia; ^4^ School of Biomedical Engineering University of New South Wales Sydney New South Wales Australia

**Keywords:** double emulsion droplets, liposomes, microfluidics, Pluronic F‐127, thermally triggered release

## Abstract

Stimuli‐responsive delivery systems, particularly thermoresponsive platforms, are becoming increasingly important for physiological applications, but their development has been limited by poor biocompatibility, slow response, and non‐scalable fabrication methods. Liposomes offer a biocompatible and adaptable foundation for this need, but no scalable approach exists to produce uniform thermosensitive liposomes that activate above 37°C. Here, we present the first scalable strategy for generating monodisperse thermoresponsive liposomes using a corona‐discharge‐ treated PDMS double‐emulsion device. Aqueous cargo‐containing cores are encapsulated within DPPC‐containing oleic acid shells, with Pluronic F‐127 added to the outer phase to stabilize the interface. Ethanol‐mediated solvent extraction transforms these shells into lipid bilayers, and the combined use of rotational mixing and surfactant‐assisted interfacial tuning reduces extraction time by more than 50% compared with prior reports while ensuring complete bilayer formation, even for thick shells. Under regulated extraction, droplets with thin lipid membranes incorporating Pluronic F‐127 undergo abrupt rupture and cargo release at ∼41°C–45°C, providing stable encapsulation at physiological temperature and sharp thermally triggered release above body temperature. Overall, this work creates the first platform capable of producing uniform thermoresponsive liposomes that undergo abrupt, super‐physiological temperature‐triggered release, providing broad potential for drug delivery, biosensing, and synthetic biology.

## Graphical abstract

Uniform thermoresponsive liposomes are generated using a scalable double‐emulsion microfluidic approach coupled with accelerated solvent extraction. The resulting liposomes remain stable at physiological temperature but undergo abrupt rupture and cargo release at 41°C–45°C, providing a versatile platform for thermally triggered controlled release in drug delivery, biosensing, and synthetic cell technologies.

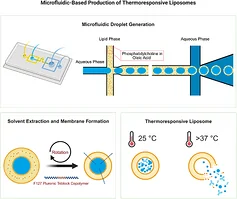

## 1 Introduction

Cancer therapy, biosensing, synthetic biology, and other biomedical applications could all benefit from controlled, site‐specific release of therapeutic payloads. Several lines of research have been explored in the literature to create materials which respond to triggers such as enzyme activity, pH, and redox gradients, or external stimuli including temperature, magnetic fields, ultrasound, or light [1, 2, 3]. Carriers have been developed at sizes spanning from nanometers to microns, depending on the application [4, 5, 6]. Among these, temperature represents perhaps one of the most interesting because it can enable precise, on‐demand payload release. Liposomes, small artificial vesicles with a lipid bilayer, are especially attractive due to their inherent biocompatibility, biodegradability, and stability [7]. To date, no scalable method for producing thermoresponsive liposomes that reliably activate above body temperature has been developed, despite the large volume of literature devoted to hyperthermia‐triggered therapeutics and stimuli‐responsive biosensing.

Liposomes have become invaluable tools in biomedical science since they were introduced in 1964 [8, 9, 10, 11, 12]. Liposomes are structurally very similar to biological cells and organelles, and their lipid encapsulation allows them to be biocompatible, biodegradable, and to mimic vital cellular functions. Depending on the materials used, it is possible to encapsulate them with both hydrophilic and hydrophobic compounds, meaning that they can be designed to carry a wide range of cargo [13, 14, 15]. In recent years, liposomes have been used in gene therapy [16], cancer therapeutics, and the development of synthetic cells [17, 18]. Liposomes are now also considered as leading candidates for the development of smart drug delivery systems, capable of fine‐tuning release mechanisms and targeting specific tissues [19]. Unilamellar liposomes are categorized based on size into small (<100 nm), large (100–1000 nm), and giant unilamellar vesicles (GUVs, >1 µm). Owing to their cell‐like dimensions, GUVs are especially valuable for applications in membrane biophysics and artificial cell models [20]. A critical requirement for the reliable use of GUVs is size uniformity.

Traditional liposome fabrication methods, such as thin‐film hydration, ethanol injection, and reverse‐phase evaporation, remain widely employed but are constrained by issues including poor size uniformity, low encapsulation efficiency, and significant material wastage. In contrast, emerging microfluidic approaches offer a powerful solution to these limitations [20] and also enable integration with lab‐on‐a‐chip platforms for real‐time monitoring, particle sorting, and downstream analysis. A notable strategy in liposome fabrication involves templating GUVs from double‐emulsion droplets. In a seminal approach by Shum et al., water‐in‐oil‐in‐water (w/o/w) droplets were generated using glass capillary devices, and subsequent solvent evaporation led to phospholipid self‐assembly into bilayers [21]. Despite successful vesicle formation, residual solvents, such as chloroform and hexane, often persisted, raising concerns about biocompatibility. To minimize solvent retention, ultrathin oil shells (<1 µm) were employed in modified designs [22, 23]. Due to the rapid evaporation of the intermediate oil layer, complete vesicle formation was often observed inside the device before droplets exited the chip. However, once the oil layer was removed, these phospholipid vesicles tended to be fragile and could not withstand transfer off‐chip, limiting their suitability for downstream manipulation [24]. Further refinement was achieved by incorporating Pluronic block copolymer surfactants into the outer aqueous phase, where they modulate interfacial energies and promote dewetting [17, 25, 26]. These surfactants not only facilitated solvent removal but also improved the stability and yield of the resulting vesicles. Their interactions with lipid bilayers have been well‐documented in several studies [27, 28]. Among them, Pluronic F‐127, a biocompatible and thermoresponsive triblock copolymer, has been shown to influence the thermal release behavior of liposomes [29].

Soft lithography has enabled the cost‐effective, flexible design of polydimethylsiloxane (PDMS)‐based microfluidic chips. One‐octanol is among the most widely used solvents for liposome formation [30, 31, 32]. In specific chip designs, such as those employing six‐way junctions, 1‐octanol forms a pocket containing excess lipids that gradually detaches from the main vesicle body within seconds to minutes after droplet formation. However, if the droplet shell is excessively thick, dewetting may be incomplete, leading to the retention of lipid‐rich pockets and inefficient vesicle production [31]. Moreover, due to the nature of the solvent‐based process, small amounts of 1‐octanol may persist within the lipid bilayer [30, 31], potentially affecting membrane stability or biocompatibility. Oleic acid is another promising solvent, offering compatibility with PDMS due to its non‐swelling effect on the polymer. In this approach, liposomes are generated as w/o/w droplets with an oleic acid middle layer and an ethanol‐containing outer phase [24, 33, 34, 35, 36]. Ethanol acts as a mild extractant, gradually removing oleic acid from the shell and promoting liposome formation through spontaneous lipid bilayer self‐assembly. While this approach avoids the use of toxic organic solvents and offers excellent biocompatibility, the extraction process remains time‐consuming. Previous studies have reported that complete oleic acid extraction and liposome formation require 15–18 h, even for double emulsions with relatively thin oleic acid shells [24, 33, 36]. Furthermore, the need for surface treatment of PDMS channels represents an additional bottleneck, as stable operation often relies on polyvinyl alcohol (PVA) coatings to tailor channel wettability, increasing fabrication complexity and reducing device accessibility [32]. In addition, lipid droplet generation in PDMS‐based oil‐in‐water systems is particularly difficult because of the strong hydrophobicity of lipids [37]. Spatially controlled plasma treatments using corona discharge systems have emerged as a promising alternative, enabling localized tuning of surface wettability within PDMS chips. This approach simplifies device fabrication, improves reliability [38], and offers the potential for more efficient liposome production in PDMS‐based microfluidic devices.

The Thermoresponsive liposomes represent a particularly attractive class of stimuli‐responsive carriers, as they undergo a gel‐to‐liquid crystalline phase transition that increases membrane permeability and triggers cargo release at a defined temperature [39]. By selecting appropriate lipid compositions, this transition can be tuned to coincide with hyperthermia (∼40°C–45°C), enabling externally controlled release [7, 40, 41]. However, the production of thermoresponsive liposomes has predominantly relied on bulk preparation methods, which often yield limited size uniformity at the nanoscale. For example, liposomes with diameters ranging from 150 to 230 nm [41] and 426 ± 346 nm (mean ± SD) [7] have been reported. In contrast, microfluidic devices enable the generation of highly monodisperse vesicles through double‐emulsion templating. Dipalmitoylphosphatidylcholine (DPPC), with a phase transition temperature of ∼41°C, is particularly well suited for heat‐triggered delivery applications. Nevertheless, most microfluidic liposome studies have focused on lipids such as DOPC, DOPG, DOPE, POPC, or DOPS [24, 30, 33, 42, 43, 44, 45], whose low transition temperatures (−18°C to −2°C) [46, 47, 48, 49, 50] may limit their suitability for thermally triggered release above physiological temperature.

**FIGURE 1:**
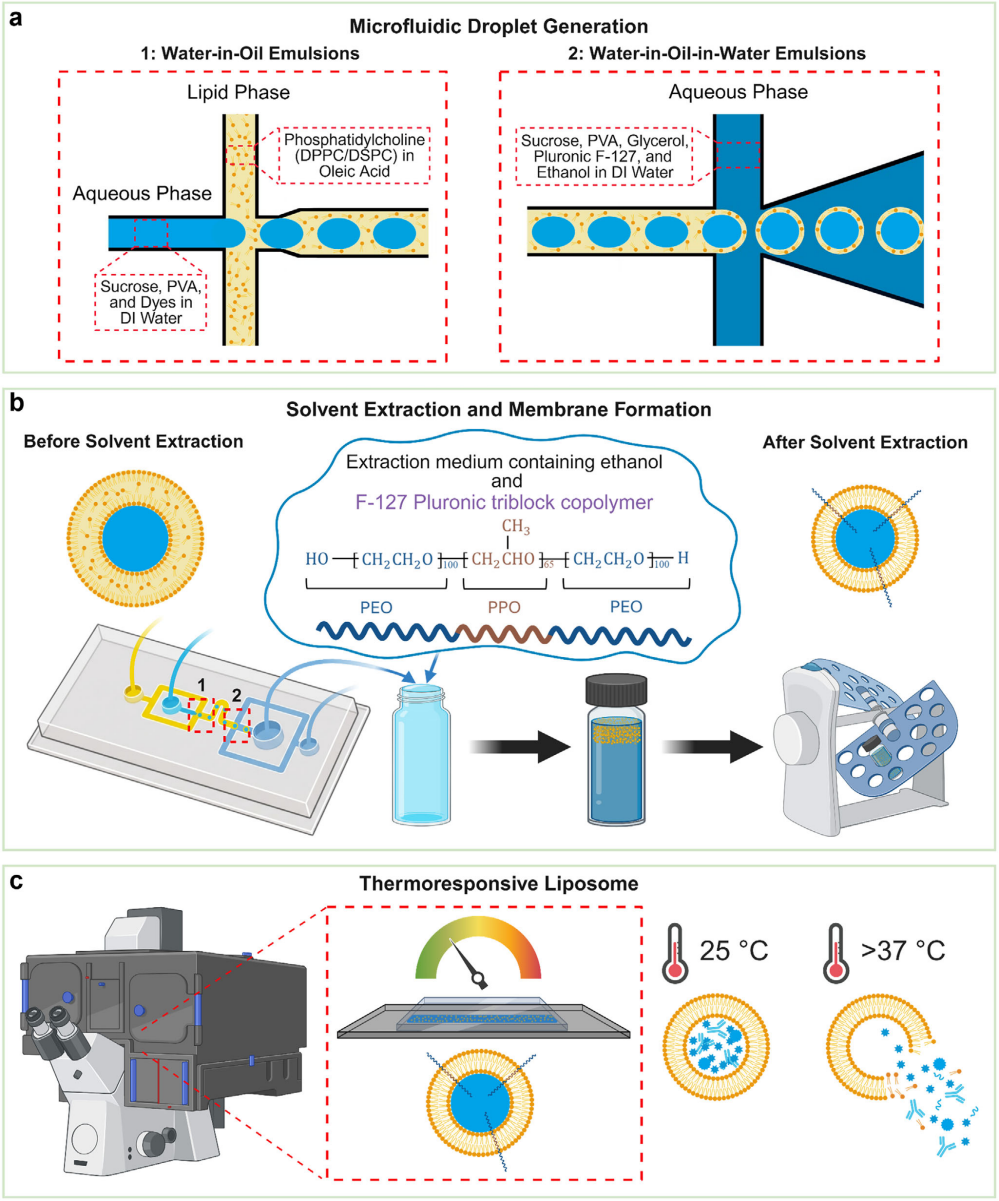
Schematic illustration of a custom microfluidic for lipid droplet fabrication and subsequent solvent extraction to form thermoresponsive liposomes. (a) Microfluidic droplet generation using lipid‐containing and aqueous phases converges in a custom microfluidic device to form highly uniform w/o/w double‐emulsion droplets. (b) Subsequent controlled solvent extraction enhanced by rotational mixing and Pluronic F127 surfactant, drives the self‐assembly of a stable lipid bilayer shell on the droplets to yield monodisperse liposomes. (c) These liposomes exhibit thermoresponsive behavior above 37°C, triggering rapid cargo release via liposome disassembly.

Despite significant progress in microfluidics and liposome engineering, a robust, scalable method for producing highly uniform microscale thermoresponsive liposomes that release their contents within a precisely defined temperature window has yet to be established. In this study, we sought to develop a controllable, scalable approach to fabricate core‐shell liposomes that respond predictably to elevated temperatures. Using a corona‐discharge‐treated PDMS double‐emulsion microfluidic device, we generated stable DPPC‐containing oleic‐acid shells without additional microchannel surface coatings (Figure 1a). By coupling ethanol‐mediated extraction with gentle rotational agitation and surfactant‐assisted interfacial modulation, we significantly accelerated bilayer formation while ensuring effective solvent removal, including in droplets with thicker shells (Figure 1b). In the presence of Pluronic F‐127, the liposomes exhibited thermoresponsive behavior, with abrupt rupture and cargo release occurring within the 41°C–45°C (Figure 1c). Together, this study establishes a robust, scalable platform for producing uniform thermoresponsive liposomes, opening new opportunities in controlled drug delivery, biosensing, and synthetic biology.

## 2 Results and Discussions

### 2.1 Microfluidic Device Design for Double‐Emulsion Droplet Formation

**FIGURE 2:**
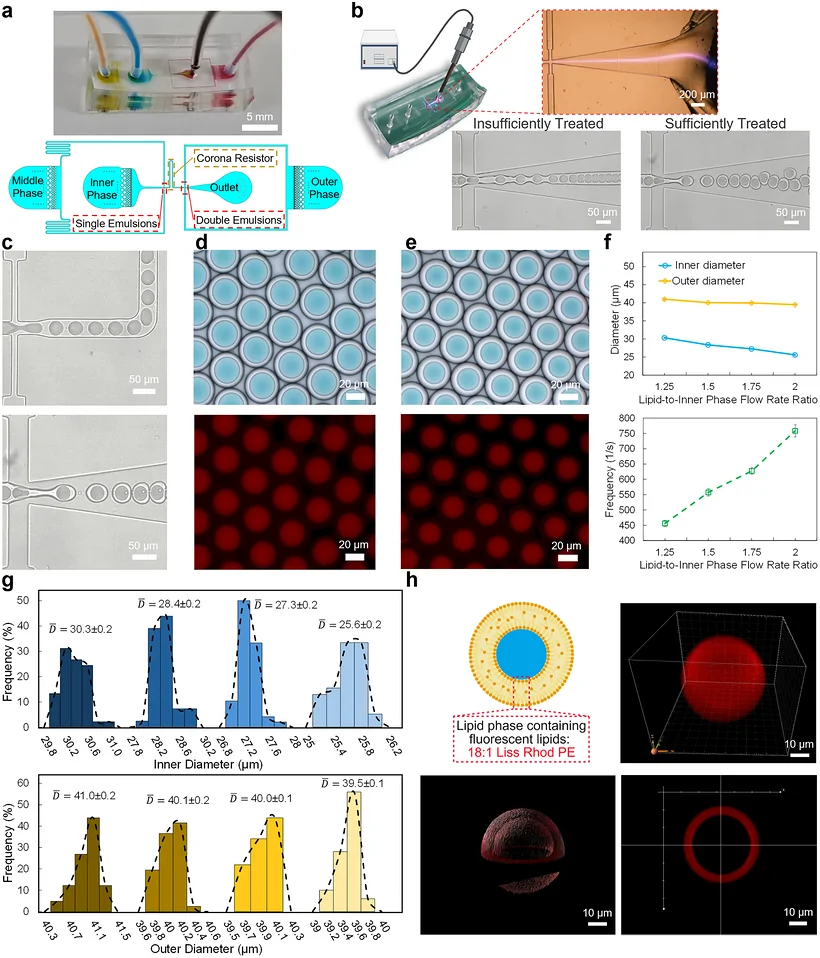
Generation and characterization of monodisperse double‐emulsion droplets. (a) Photograph (top) and schematic (bottom) of the microfluidic device. Shallow channels (∼30 µm) for the inner (blue) and middle (yellow) phases, followed by encapsulation in the outer phase (red) within deeper channels (∼50 µm). (b) Corona treatment of the PDMS‐glass chip. The electrode is applied at the outlet; the inset shows the plasma discharge. Bottom panels show the outlet under insufficient and sufficient treatments. (c) Bright‐field images of w/o (top) and w/o/w (bottom) emulsions at a lipid‐to‐inner phase flow rate ratio of 1.25. (d, e) Bright‐field (top) and fluorescence (bottom) images of droplets produced at lipid‐to‐inner phase flow‐rate ratios of (d) 1.25 and (e) 2.0. f) Inner and outer diameters (top) and production frequency (bottom) as functions of the lipid‐to‐inner phase flow rate ratio (inner phase flow rate = 0.4 µL min^−1^). g) Frequency histograms of inner (top) and outer (bottom) droplet diameters for different flow‐rate ratios. h) Confocal fluorescence imaging of a representative droplet with fluorescent lipids (18:1 Liss Rhod PE), showing a spherical morphology with a well‐defined aqueous core and lipid shell.

Here, we developed a microfluidic‐based strategy to fabricate highly monodisperse w/o/w double‐emulsion droplets using a flow‐focusing geometry, in which a lipid phase containing oleic acid is positioned between two aqueous phases (Figure 2a). The outer aqueous phase contains sucrose, PVA, glycerol, Pluronic F‐127, and ethanol, while the inner aqueous phase contains sucrose, PVA, and dyes. The chip design (Figure 2a) includes two sequential flow‐focusing junctions: the first junction forms water‐in‐oil (w/o) emulsions by combining the inner aqueous phase with a lipidic middle phase (DPPC dissolved in oleic acid) (Movie S1, Supporting Information), while the second junction shears the w/o droplets with the outer aqueous phase to generate w/o/w double emulsions (Movie S2, Supporting Information). Due to PDMS's inherent hydrophobicity and the strong hydrophobicity of lipids, the device's outlet region required surface modification to enable stable droplet formation. This was achieved rapidly and cost‐effectively using a corona‐discharge plasma system that locally tuned the outlet surface wettability (Figure 2b). The treatment created distinct hydrophobic and hydrophilic regions within the device, which are essential for the controlled generation of water‐in‐oil and oil‐in‐water droplets. To regulate interfacial energies and facilitate droplet formation, the surfactant Pluronic F‐127 was introduced into the outer aqueous phase, thereby lowering the interfacial tension between the middle and outer phases. However, this adjustment also altered the interfacial energy balance between the generated droplets and the surrounding medium. Consequently, at a concentration of 5% (w/v) Pluronic F‐127, droplet rupture was occasionally observed, particularly when the shells were thin. These observations indicate a trade‐off between enhancing droplet generation and preserving structural stability as the surfactant concentration in the outer phase is varied. To mitigate this issue, a concentration of 3% (w/v) Pluronic F‐127 in the outer aqueous phase was found to be sufficient to maintain stable double‐emulsion formation when the lipid concentration in oleic acid is maintained at 2 mg/mL. Glycerol improved droplet stability, while PVA helped prevent coalescence by stabilizing the interfaces [24, 33]. Notably, the lipid phase used in the present study did not contain cholesterol, a component commonly incorporated into liposomal formulations to improve membrane stability, increase lipid packing density, and reduce bilayer permeability. However, cholesterol concentration is critical, as low levels can introduce membrane defects and increase leakage, while excessively high amounts may overly restrict release [51].

Figure 2c presents bright‐field images capturing the first and second flow‐focusing regions, where single and double emulsions are formed at a lipid‐to‐inner phase flow rate ratio of 1.25. When the outer aqueous phase shears the w/o droplets, a small fraction of the lipid phase separates near the junction. This separation arises from an imbalance between viscous and interfacial forces during droplet pinch‐off, where excessive shear from the continuous phase leads to partial detachment of the lipid layer from the droplet interface. Figure 2d,e show both bright‐field (upper) and fluorescence (lower) microscopy images of droplets at lipid‐to‐inner flow rate ratios of 1.25 and 2, respectively. At the higher flow rate ratio, the shell thickness increases while the core size decreases, reflecting altered breakup dynamics at the first flow‐focusing junction. At the lower flow rate ratio (Figure 2c), the droplets exhibit well‐defined, centred aqueous cores with relatively thin lipid shells, suggesting production in a stable dripping regime. At a lipid‐to‐inner phase flow rate ratio of 2.0, although the droplets remain discrete, the observed changes in morphology and spacing suggest a transition in the second flow‐focusing region toward a regime influenced by geometric confinement and increased viscous forces, potentially approaching jetting, though still within the dripping domain (Movie S3, Supporting Information). A blue dye was added to the inner phase to clearly distinguish the core‐shell structure. The fluorescent images confirm successful encapsulation of the inner aqueous phase within the lipid‐oleic acid shell.

Figure 2f plots the relationship between lipid‐to‐inner phase flow rate ratio and both droplet dimensions and production frequency. Increasing the lipid flow rate results in a thicker shell, providing tunable, precise control over the amount of lipid encapsulated in each droplet. The droplet generation frequency increases with higher lipid‐to‐inner‐phase flow‐rate ratios. Droplet size distributions at different flow‐rate ratios are presented in Figure 2g, showing narrow distributions for both the inner and outer diameters. For instance, at a lipid‐to‐inner‐phase flow‐rate ratio of 1.25, the mean inner and outer diameters are 30.3 ± 0.2 µm and 41.0 ± 0.2 µm, respectively. This high uniformity facilitates the subsequent solvent extraction step in liposome production, as the droplet shells have nearly identical thicknesses, enabling complete solvent extraction across all droplets simultaneously. The droplet morphology was further analyzed using fluorescently labelled lipids under confocal laser scanning microscopy, confirming a distinct separation between the aqueous core and the lipid‐rich shell, with slight interfacial mixing and a well‐defined core‐shell structure (Figure 2h and Movie S4, Supporting Information). Three‐dimensional confocal reconstruction reveals the overall volume and spherical morphology of a representative droplet, while the corresponding cross‐sectional view highlights both the inner and outer shell interfaces, clearly delineating the core‐shell architecture.

### 2.2 Solvent Extraction and Membrane Formation

**FIGURE 3:**
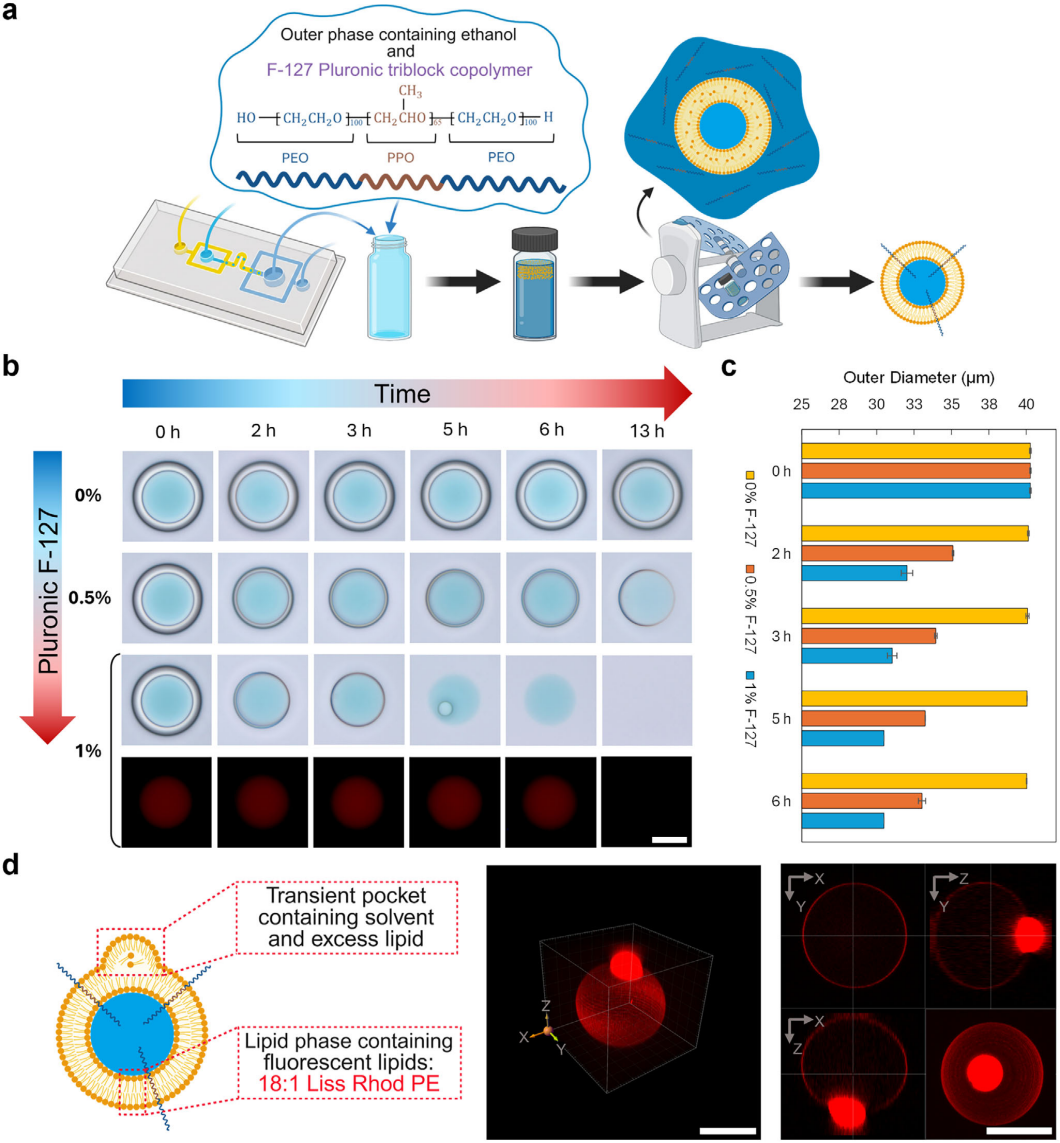
Extraction of oleic acid from double‐emulsion droplets and bilayer membrane formation. (a) Schematic of the extraction process: w/o/w droplets with lipid‐oleic acid shells were dispersed in an ethanol‐containing aqueous phase with Pluronic F‐127, leading to solvent removal and bilayer self‐assembly. (b) Time‐lapse bright‐field images show intact shells without surfactant (top), partial extraction with 0.5% Pluronic F‐127 (middle), and complete extraction within 6 h at 1% Pluronic F‐127 (bottom). Fluorescence images confirm the encapsulation of the red dye after bilayer formation. (c) Quantification of droplet size highlights shell thinning and size reduction with increasing Pluronic F‐127 concentration. (d) Confocal fluorescence imaging of droplets. The lipid phase contained fluorescent lipid 18:1 Liss Rhod PE. 3D reconstruction and X‐Y‐Z cross‐sections reveal a transient pocket containing solvent and excess lipid, along with bright peripheral fluorescence indicating the formation of the lipid membrane. The droplet generation was performed at a lipid‐to‐inner‐phase flow‐rate ratio of 1.25. Scale bars, 20 µm.

The extraction of oleic acid from the shells of w/o/w double‐emulsion droplets was investigated in an aqueous outer phase containing 14% (v/v) ethanol, which acted as the extracting agent due to its high solubility for oleic acid (Figure 3a). To ensure osmotic balance during droplet formation, storage, and extraction, the composition of the inner aqueous phase was adjusted to match that of the outer phase, thereby preventing swelling or shrinkage. Initially, owing to the lower density of the oleic acid phase compared with the surrounding aqueous medium, the droplets floated near the surface. Gentle rotation was applied to enhance contact between the shells and the ethanol‐rich medium, promoting shell‐medium interaction and improving extraction efficiency. Surfactants in the outer phase further reduced interfacial tension and stabilized the droplets during extraction. Pluronic F‐127, a triblock copolymer composed of poly(ethylene oxide)‐poly(propylene oxide)‐poly(ethylene oxide) (PEO_100_‐PPO_65_‐PEO_100_), not only facilitated solvent removal but also interacted with the lipid bilayers. Notably, the PPO segment is long enough (>40 repeat units) to span the lipid bilayer, making a membrane‐spanning mechanism the most plausible mode of interaction between this copolymer and lipid molecules [52].

The droplets generated at a lipid‐to‐inner phase flow rate ratio of 1.25, which exhibited the smallest shell thicknesses, were selected for solvent extraction. Bright‐field images in Figure 3b illustrate the time‐dependent effect of Pluronic F‐127 concentration on the extraction process. In the absence of surfactant (first row), the oil shell remained intact, and no extraction occurred. At 0.5% Pluronic F‐127 (second row), partial extraction was observed, with a noticeable reduction in shell thickness but incomplete bilayer formation. With 1% Pluronic F‐127 (third row), nearly complete extraction was achieved within 6 h, substantially faster than previously reported times for similar systems [24, 33]. Fluorescence images in the bottom row of Figure 3b confirm successful encapsulation of red dye (Alexa Fluor 647‐labelled dextran) within the aqueous core after membrane formation. This approach proved effective across droplets with different initial shell thicknesses. A blue dye was added to the aqueous core to improve visualization, as the final lipid membrane is ultrathin and difficult to resolve under bright‐field microscopy.

During the extraction, ethanol gradually removed oleic acid from the shell, thinning it until the inner and outer lipid monolayers came into contact and self‐assembled into a continuous bilayer membrane. This rearrangement is driven by favorable hydrophilic interactions between lipid head groups and the aqueous environment, as well as van der Waals forces between hydrophobic tails. Any remaining oil, being less dense than the aqueous core, often formed a small pocket before disappearing, similar to the mechanism described for chloroform‐hexane systems [23]. This transient solvent pocket, together with excess lipids, can be visualized when fluorescently labelled lipids are incorporated into the lipid phase, as shown in Figure 3d and Figure S1, Supporting Information. In Figure 3d, confocal fluorescence microscopy was used to image these intermediate droplets during extraction. The lipid phase contained fluorescent lipid 18:1 Liss Rhod PE, enabling visualization of lipid‐rich regions. The 3D reconstruction and orthogonal X‐Y‐Z cross‐sections reveal bright peripheral fluorescence outlining the forming lipid membrane, along with localized internal fluorescence corresponding to transient solvent pockets and excess lipid domains. These observations confirm dynamic lipid rearrangement and the presence of intermediate structures during oleic acid removal, preceding complete bilayer formation. Due to variations in initial shell thickness, some droplets reached the fully extracted membrane faster than others, resulting in a mixture of partially and fully extracted shells or occasional droplet breakage. Quantitative analysis of droplet size (Figure 3c) shows that surfactant concentration influenced the outer diameter. Droplets without surfactant maintained their size, whereas those exposed to 0.5% and 1% Pluronic F‐127 decreased in size, consistent with progressive extraction and shell thinning. The fabricated liposomes maintained their structural integrity after two weeks of storage at room temperature (Figure S2, Supporting Information).

### 2.3 Thermoresponsive Behavior and Release Functionality of Liposomes

Now that uniform droplets and an efficient extraction process have been developed, the next step is to test their thermoresponsive behavior. To model a variety of potential cargo sizes, molecular dyes and larger polymeric microbeads were introduced into the droplet cores.

**FIGURE 4:**
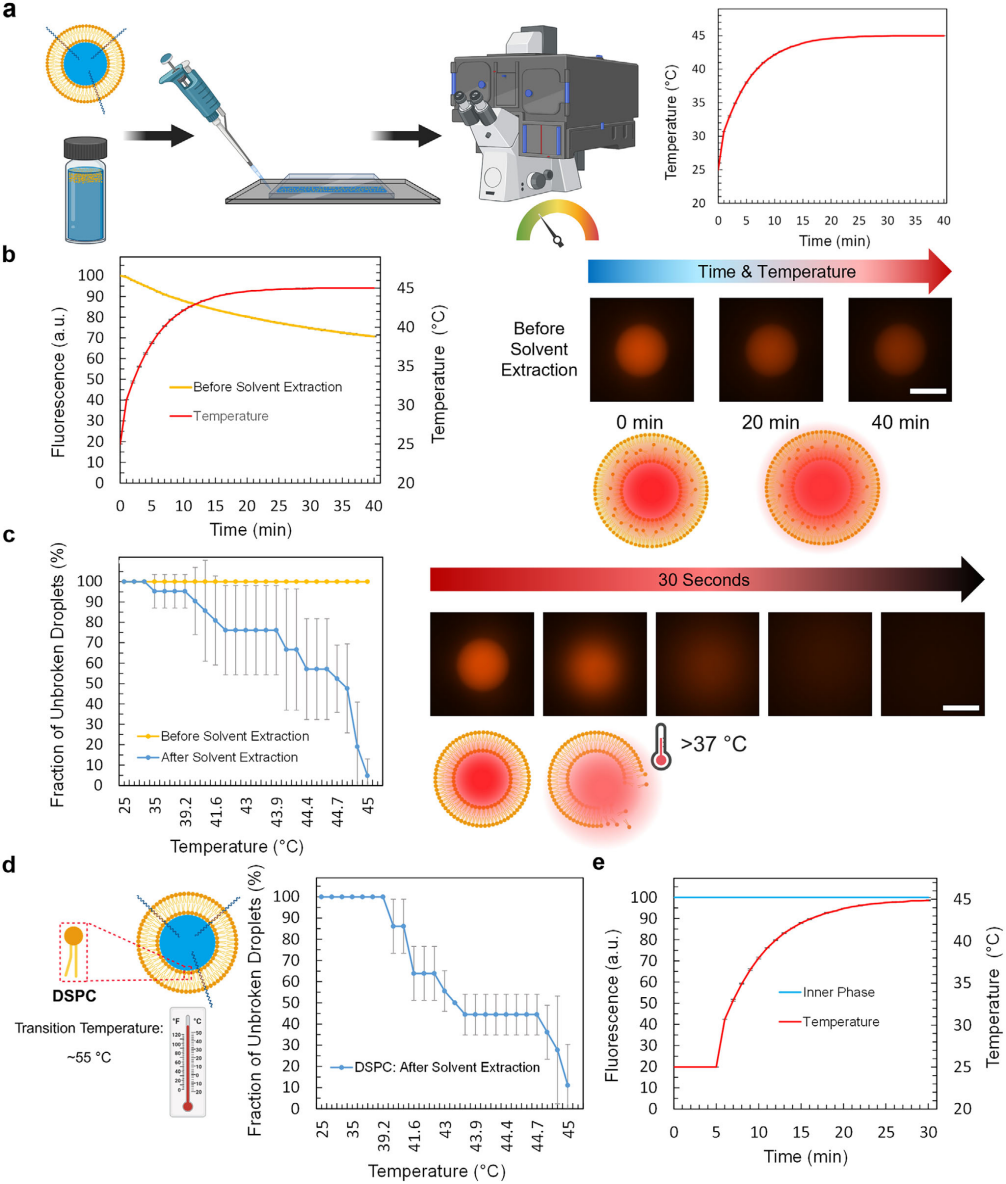
Thermal‐triggered release of liposomes. (a) Schematic of droplet deposition in an observation chamber, fluorescence imaging, and temperature evolution during heating. (b) Fluorescence intensity of liposomes before solvent extraction as a function of time, showing a moderate (∼30%) increase in payload release at elevated temperatures. Although heating enhances release, the overall change remains limited. Representative time‐lapse fluorescence images are shown. (c) Fraction of unbroken droplets as a function of temperature (mean ± SD, *n* = 3 replicates), demonstrating enhanced thermal sensitivity after solvent extraction, accompanied by a schematic illustrating liposome membrane behavior above ∼37°C. (d) Thermal response of DSPC‐based liposomes (mean ± SD, *n* = 3 replicates). (e) Control experiments confirming dye stability. Scale bars: 20 µm.

The thermal response and payload release of the prepared liposomes were systematically evaluated to assess their stability and temperature‐triggered behavior. Figure 4a shows the experimental setup, in which droplets containing the red fluorescent dye encapsulated in their cores were transferred to an observation chamber and subjected to controlled heating, with fluorescence intensity monitored. The temperature profile indicates a gradual increase from room temperature to 45°C. In Figure 4b, the release behavior of droplets with no solvent extraction (oil retained in the shell) is evaluated. For droplets retaining solvent, dye release was relatively limited, reflecting partial permeability of the shell upon heating.

Figure 4c presents the case of fully extracted droplets or droplets with transient solvent pockets, where the membranes consisted of lipid bilayers containing incorporated Pluronic. Heating beyond the DPPC transition temperature resulted in a marked loss of droplet integrity. The fraction of unbroken droplets decreased sharply above ∼41°C–42°C, indicating structural failure (Movie S5, Supporting Information). This temperature range is consistent with the main transition temperature of DPPC (∼41°C), at which the bilayer transitions from a gel to a fluid phase, increasing lipid‐tail mobility, introducing packing defects, and enhancing permeability [7, 53]. Importantly, even after diluting the outer phase with F‐127‐free medium to substantially reduce free surfactant concentration, droplets still ruptured upon heating. This suggests that the instability is mainly due to membrane‐incorporated Pluronic rather than to residual surfactant in the bulk solution.

To further investigate the behavior, additional experiments were performed using distearoylphosphatidylcholine (DSPC) liposomes, which have a higher main transition temperature (∼55°C). As shown in Figure 4d, droplet rupture still occurred well below the DSPC transition temperature, indicating that the disruption might not be attributed solely to the lipid phase change. Previous studies on nanoscale thermosensitive liposomes have shown that the temperature window for leakage can be shifted by modifying the lipid composition. For example, incorporation of lipids with higher melting temperatures, such as DSPC, can increase the leakage temperature range relative to DPPC‐based systems [7, 41]. In such systems, gradual permeability enhancement is typically observed above the lipid phase‐transition temperature. In the present work, however, micron‐sized liposomes with well‐defined core‐shell architectures exhibited abrupt rupture within a relatively narrow temperature range (∼41°C–45°C), regardless of whether DPPC or DSPC‐containing membranes were used, despite the substantially higher gel‐to‐liquid crystalline transition temperature of DSPC (∼55°C). This observation suggests that the thermal response cannot be explained solely by the lipid phase transition. One possible contributing factor is the interaction of Pluronic F‐127 with the lipid membrane. Interactions between Pluronic block copolymers and lipid bilayers have been reported previously [27, 28]. Pluronic F‐127 is a thermoresponsive triblock copolymer composed of poly(ethylene oxide)‐poly(propylene oxide)‐poly(ethylene oxide) (PEO_100_‐PPO_65_‐PEO_100_), and has been reported to influence the thermal release behavior of liposomes [28, 29]. The hydrophobic PPO block is sufficiently long to potentially span or insert into lipid bilayers, making membrane association plausible [52]. In addition, Pluronic F‐127 undergoes temperature‐dependent structural rearrangement, where hydrophobic PPO segments increasingly associate while hydrophilic PEO chains extend into the aqueous phase [54]. Accordingly, we hypothesise that the observed membrane rupture may arise from the combined effects of lipid membrane softening and thermoresponsive Pluronic‐lipid interactions, rather than from lipid phase transition alone. The persistence of rupture behavior in DSPC‐containing liposomes supports the possibility that Pluronic F‐127 contributes substantially to membrane destabilization at elevated temperatures. However, direct experimental confirmation of Pluronic incorporation within the membrane was not performed in the present study, and therefore, the proposed mechanism should be interpreted as a hypothesis supported by the observed thermal behavior. Control experiments (Figure 4e) confirmed that neither excitation light nor heating alone altered the dye's fluorescence, ruling out photobleaching or dye degradation as artifacts.

Overall, the results from both DPPC and DSPC systems indicate that lipid phase transitions may influence membrane permeability to some extent, while thermoresponsive restructuring of Pluronic F‐127 within the membrane may also contribute significantly to droplet rupture. In summary, complete extraction yields membranes that undergo abrupt structural collapse when heated above physiological temperatures. These findings suggest that the observed thermal sensitivity likely arises from the combined effects of lipid composition, residual solvents, and Pluronic‐mediated destabilization.

### 2.4 Encapsulation and Release of Large Payloads

**FIGURE 5:**
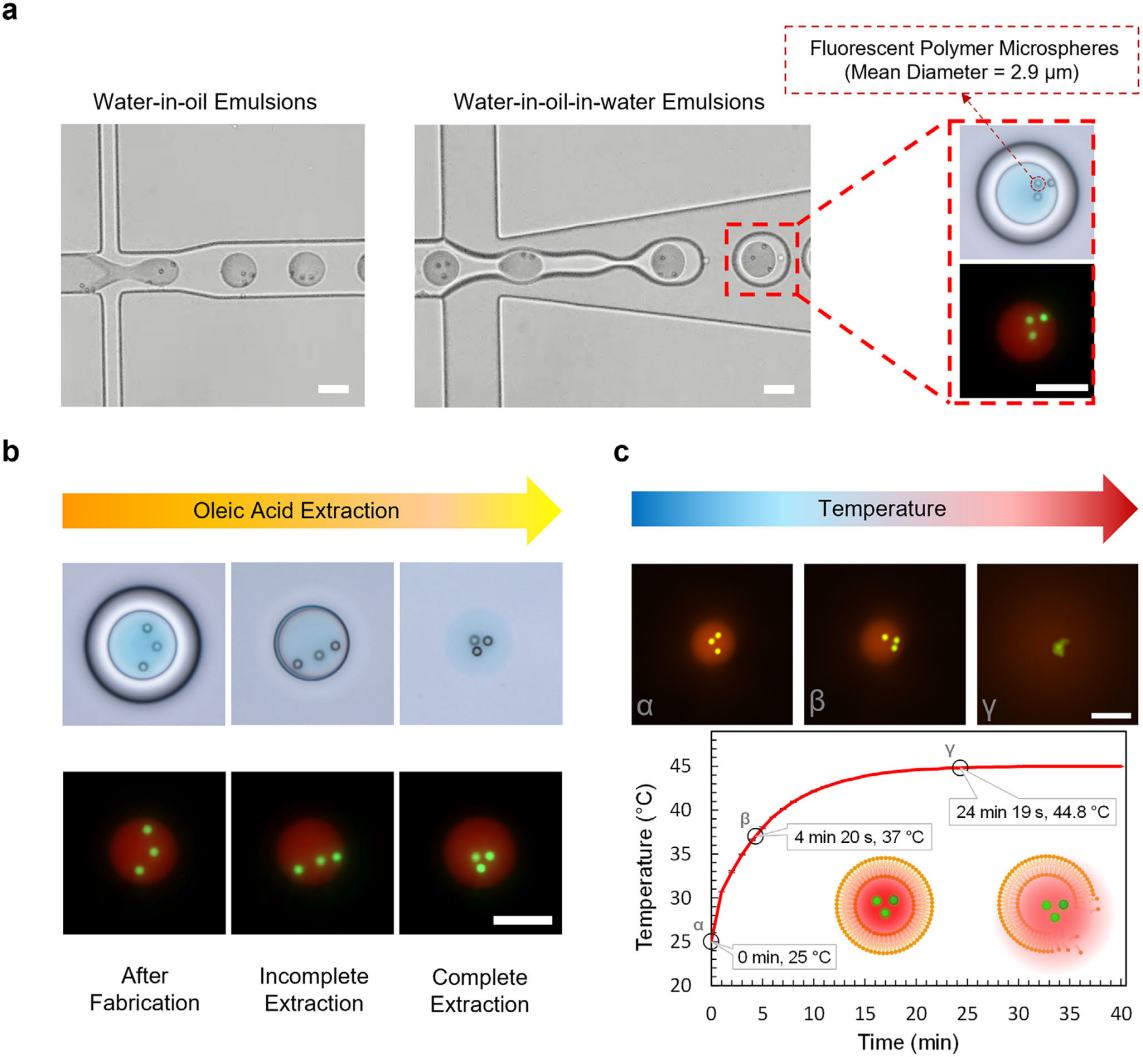
Encapsulation and release of large fluorescent microspheres (2.9 µm) in liposomes. (a) Water‐in‐oil (w/o) and water‐in‐oil‐in‐water (w/o/w) emulsions were formed at the primary and secondary junctions, with bright‐field and fluorescence images confirming microsphere encapsulation. (b) Oleic acid was extracted from the shell, yielding stable liposomes retaining the microspheres. (c) Thermal sensitivity of the membrane caused liposome rupture and release of the entrapped beads. Scale bars: 20 µm.

To demonstrate the system's capability to encapsulate and release large cargos, fluorescent polymer microspheres (2.9 µm) were entrapped within DPPC‐based liposomes. In Figure 5a, w/o emulsions were first formed at the primary flow‐focusing junction and subsequently converted into w/o/w double emulsions at the secondary junction. Bright‐field and fluorescence images confirmed that multiple microspheres were encapsulated inside the aqueous core surrounded by a DPPC‐containing shell. In Figure 5b, the oleic acid extraction step is shown, in which the removal of the organic solvent resulted in the formation of stable liposomes that retained the encapsulated microspheres. In Figure 5c, the thermal sensitivity of the membrane is demonstrated, where elevated temperature caused rupture of the liposome and release of the entrapped beads. Collectively, these results show that efficient encapsulation was achieved and that the system can accommodate and release particles much larger than small molecules or dyes, consequently extending its possible applications.

## 3 Conclusion

This study shows a rapid, biocompatible, and tunable microfluidic strategy for fabricating thermoresponsive liposomes that tackles limitations in existing systems, including poor uniformity, slow response kinetics, and inefficient solvent‐extraction methods. The approach combines double‐emulsion droplet generation in a PDMS device with an ethanol‐based oleic acid extraction method, enhanced by rotational mixing and surfactant‐mediated interfacial tuning. These modifications overcome previous challenges in oleic acid solvent‐based protocols by reducing extraction time by more than 50% while achieving complete bilayer formation, even in droplets with thick shells, limitations not previously resolved in the literature.

A critical enabling factor for reliable w/o/w emulsion formation is the careful management of surface wettability within the inherently hydrophobic PDMS microfluidic device. Given both PDMS's hydrophobicity and the strong hydrophobicity of lipid phases, stable double‐emulsion formation requires precise, localized surface modification at the outlet. Conventional approaches rely on time‐consuming and often unstable chemical coatings. Here, we utilized a rapid, cost‐effective corona‐discharge plasma treatment to locally tune surface wettability, creating spatially distinct hydrophobic and hydrophilic regions within a single device. This selective wettability patterning is essential for the sequential formation of water‐in‐oil and oil‐in‐water droplets. It provides a robust, scalable alternative to conventional coating methods, significantly improving device reliability and throughput.

Droplets with fully extracted membranes exhibited abrupt structural collapse at temperatures above normal body temperature, highlighting the importance of solvent removal in establishing functional bilayer membranes. This feature is attractive for biomedical applications, particularly hyperthermia‐assisted drug delivery, where release can be confined to diseased tissue exposed to mild heating while minimising premature leakage under normal conditions.

Experiments with both DPPC and DSPC liposomes reveal that while lipid phase transitions can modulate permeability, droplet rupture may also be strongly influenced by the thermoresponsive behavior of Pluronic F‐127 within the membrane. Pluronic F‐127 not only facilitates solvent extraction by reducing interfacial tension, along with consequently accelerating oleic acid removal, but may also interact directly with lipid bilayers. Notably, liposome rupture persists even when the free surfactant in the outer aqueous phase is substantially diluted, suggesting that membrane‐associated Pluronic, rather than bulk surfactant, may play a significant role in thermal destabilization. These findings suggest that surfactant‐lipid interactions could constitute an additional, tunable design parameter for engineering thermoresponsive membranes, independent of lipid composition alone.

Compared with conventional bulk‐phase liposome fabrication techniques, the microfluidic approach provides tighter control over size distribution, reduces reagent consumption, and allows straightforward integration with downstream on‐chip analytical or functional assays. The successful encapsulation of both small‐molecular‐weight dyes and micron‐scale polymeric particles highlights the platform's adaptability and demonstrates its ability to accommodate cargos spanning a broad size spectrum. Together, these findings support the use of this system as a scalable and tunable strategy for generating thermoresponsive liposomes with well‐defined structural and thermal characteristics, making it relevant to applications in drug delivery, biosensing, and artificial cell research. Moreover, the design principles described here could be adapted to create multi‐responsive vesicles, for example, by incorporating additional functional materials or nanoparticle components, thereby enabling dual‐triggered or magnetically assisted release mechanisms.

Overall, this study presents a practical and scalable framework for the production of uniform thermoresponsive liposomes using PDMS‐based microfluidics, coupled with ethanol‐mediated solvent extraction and Pluronic‐enhanced membrane sensitivity. By dealing with continuing challenges in solvent removal, size uniformity, and regulated thermal activation, the approach delivers a beneficial route to the development of customizable liposomal systems with potential clinical and biomedical relevance.

## 4 Experimental Section

### 4.1 Fabrication of PDMS Microfluidic Devices

The PDMS microfluidic device was fabricated using a two‐layer soft lithography method. The microchannel layout was designed using AutoCAD software (Autodesk, USA) and patterned onto a 3‐inch silicon wafer using a direct‐write optical lithography system (MicroWriter ML3, Durham Magneto Optics, UK). The master mold comprised two layers of SU‐8 2025 photoresist (MicroChem, USA). The first layer, approximately 30 µm in height, featured a hydrophobic flow‐focusing geometry with channel widths of ∼32 µm for the inner aqueous phase and ∼14 µm for the middle lipid phase, forming water‐in‐oil droplets. The second layer, ∼50 µm deep, was designed for double‐emulsion formation via a hydrophilic flow‐focusing junction, with both the outer‐phase and single‐emulsion inlet channels having widths of ∼46 µm. The corona resistor, a serpentine channel connecting the two flow‐focusing regions, confined the area treated by corona discharge. PDMS (Sylgard 184, Dow, USA) was mixed in a 10:1 base‐to‐curing‐agent ratio, degassed under vacuum, and poured over the mold. After curing at 70°C, the PDMS layer was peeled off, and access ports were created using a 1.2 mm biopsy punch. Both the patterned PDMS and a cleaned glass slide were treated with oxygen plasma (PDC‐002, Harrick Plasma, USA) and bonded. The bonded assembly was then baked at 90°C for 10 min to reinforce adhesion (Figure S3, Supporting Information). A handheld corona treater (BD‐20AC, Electro‐Technic Products, USA) was used to modify the wettability of the deeper channel layer selectively. The electrode tip was inserted into the chip outlet and positioned in contact with the glass substrate, with the discharge power and penetration depth carefully adjusted so that the corona discharge treatment reached only the end of the deep channel. The discharge duration was tightly controlled to avoid damage to the microchannels, ensuring stable double‐emulsion generation.

### 4.2 Preparation of Lipid and Aqueous Phases

To prepare the lipid phase, 1,2‐dipalmitoyl‐sn‐glycero‐3‐phosphocholine (DPPC) (734.06 Da, Avanti Polar Lipids) was dissolved in chloroform in a glass vial, the solvent allowed to evaporate, and then oleic acid was added to achieve a final DPPC concentration of 2 mg/mL in oleic acid. When needed, the fluorescent lipid 18:1 Liss Rhod PE (1301.71 Da, Avanti Polar Lipids) was added to the lipid phase to enable visualization of the lipid shell. The mixture was gently vortexed and sonicated at temperatures below 37°C to ensure complete lipid dissolution in the oil phase.

The outer aqueous phase was prepared by dissolving sucrose (0.6 M), PVA (1% (w/v), 30 000–70 000 Da), ethanol (14% (v/v)), glycerol (10% (v/v)), and Pluronic F‐127 (3% (w/v)) in deionized water. To maintain osmotic balance during droplet formation, the inner aqueous phase was formulated to match the osmolarity of the outer phase, containing sucrose (0.6 M) and PVA (1% (w/v)). For imaging purposes, a blue food dye and a fluorescent tracer, Alexa Fluor 647‐labelled dextran (10 000 Da, Thermo Fisher Scientific), were added to the inner phase. All aqueous mixtures were vortexed until visually clear. The extraction medium was formulated with 0.6 M sucrose, 1% (w/v) PVA, 14% (v/v) ethanol, 10% (v/v) glycerol, and 0%–1% (w/v) Pluronic F‐127 in deionized water to facilitate oleic acid removal while maintaining droplet integrity during solvent extraction. Chemicals were sourced from Sigma‐Aldrich unless stated otherwise.

### 4.3 Double Emulsion Droplet Generation

Double emulsion droplets were generated using a PDMS‐based microfluidic device equipped with three inlets for introducing the inner aqueous, the middle lipid, and the outer aqueous phases, along with a single outlet for droplet collection. All phases were infused into the device using syringe pumps (Cole‐Palmer and Chemyx, USA), enabling accurate and stable control of flow rates. The outlet was connected to a collection vial via flexible tubing for subsequent analysis and processing (Figure S4, Supporting Information). Flow parameters were carefully optimized to ensure the production of uniform, monodisperse double emulsion droplets throughout the operation (Movie S6, Supporting Information).

### 4.4 Solvent Extraction Procedure

Approximately 40 µL (corresponding to ∼250 000 droplets) of the collected double emulsion output was transferred into a glass vial containing 2 mL of the extraction medium. Ethanol gradually removed oleic acid from the droplet shell into the surrounding medium. As the oleic acid diffused out, a concentrated layer of phospholipids remained around the aqueous core, promoting the spontaneous formation of a lipid bilayer. Due to the density difference between the oil shell and the aqueous medium, the droplets remained near the surface of the extraction solution. At room temperature, gentle rotation at 1 RPM was applied to enhance contact between the ethanol‐rich medium and the shell containing oleic acid, thereby improving extraction efficiency. The surfactants present in the medium further reduced interfacial tension and stabilized the droplets during the process.

### 4.5 Image Acquisition and Processing

Droplet formation was monitored in real time using an inverted microscope (Eclipse TE2000‐E, Nikon, Japan) equipped with a high‐speed camera (Phantom VEO‐610, Vision Research, USA) and a 20×/0.45 Plan Fluor objective lens, operating at approximately 12 000 frames per second to capture rapid droplet dynamics. Bright‐field and fluorescence images of the droplets were acquired using an inverted fluorescence microscope (Olympus IX73, Olympus, Japan) fitted with a DP80 digital camera and appropriate filter sets. A 40×/0.6 LUCPlanFLN objective lens was used for high‐resolution imaging. A z‐stack (3D) image dataset of the droplets was acquired using a laser‐scanning confocal microscope (Zeiss LSM 980) operated with Zen Blue software. The dye fluorescence was excited with a 561 nm (0.09%) laser with a pixel dwell time of 2.17 µs, and emission was collected in the 561–693 nm range using a GaAsP‐PMT detector. A C‐Apochromat 40×/1.20 W Korr FCS objective lens was used for image acquisition. The pixel size was set to 0.114 × 0.114 µm, and the separation between z‐planes was 0.33 µm, ensuring proper Nyquist sampling of the system's point spread function. The droplet z‐stack datasets were then imported into Imaris (Bitplane) for reconstruction, rendering, and video generation.

### 4.6 Thermal Sensitivity Analysis

To evaluate the liposomes' thermal sensitivity, we used an inverted fluorescence microscope (Eclipse Ti2, Nikon, Japan) equipped with a 20×/0.75 Plan Apo VC objective lens. The microscope features an Okolab temperature‐controlled enclosure that recirculates warm air to maintain precise thermal conditions, with a range of 20°C–50°C. Time‐lapse fluorescence videos were recorded and analyzed in Fiji to assess the extent of payload release as a function of temperature.

## Conflicts of Interest

The authors declare no conflicts of interest.

## Supporting information




**Supporting File 1**: smll74496‐sup‐0001‐SuppMat.docx.

**Supporting File 2**: smll74496‐sup‐0002‐MovieS1.mp4.

**Supporting File 3**: smll74496‐sup‐0003‐MovieS2.mp4.

**Supporting File 4**: smll74496‐sup‐0004‐MovieS3.mp4.

**Supporting File 5**: smll74496‐sup‐0005‐MovieS4.mp4.

**Supporting File 6**: smll74496‐sup‐0006‐MovieS5.mp4.

**Supporting File 7**: smll74496‐sup‐0007‐MovieS6.mp4.

## Data Availability

The data that support the findings of this study are available on request from the corresponding author. The data are not publicly available due to privacy or ethical restrictions.
