# Supplementary material for: Uniform Microscale Thermoresponsive Liposomes for Controlled Release Above Body Temperature
Source: Small. 2026 Jul 11;22(48):e74496. doi: 10.1002/smll.74496 (PMC13509117; doi:10.1002/smll.74496)
Supplement: Supplementary file 1 [file SMLL-22-e74496-s007.docx]

**Uniform Microscale Thermoresponsive Liposomes for Controlled Release Above Body Temperature**

S. Morteza Mousavi ^a, b^, Yang Zhang ^a^, Elvis Pandzic ^c^, David W. Inglis ^b^, Megan S. Lord ^d^_,_ Robert A Taylor ^a^, Ming Li ^a, b,^ ^[1]*^

^a^ School of Mechanical and Manufacturing Engineering, University of New South Wales, Sydney, NSW, 2052, Australia.

^b^ School of Engineering, Macquarie University, Sydney, NSW, 2109, Australia.

^c^ Katharina Gaus Light Microscopy Facility, Mark Wainwright Analytical Centre, University of New South Wales, Sydney, NSW, 2052, Australia.

^d^ School of Biomedical Engineering, University of New South Wales, Sydney, NSW, 2052, Australia.

**Supplementary Information Overview**

This Supplementary Information provides additional experimental details and characterization data that support the findings presented in the manuscript titled “Uniform Microscale Thermoresponsive Liposomes for Controlled Release Above Body Temperature”. The supplementary figures collectively illustrate the fabrication process of the microfluidic device, the generation of double emulsions, the morphological features, and the stability of the resulting liposomes.

**Figure S1** illustrates the ethanol-mediated extraction of oleic acid from the droplet shells. The schematic and time-lapse microscopy images capture the dynamic thinning of the shell and the transient formation of solvent pockets during the transformation into lipid bilayer membranes.

**Figure S2** presents bright-field and fluorescence microscopy images of liposomes after 7 and 14 days of storage in solution at room temperature. The images demonstrate the long-term structural stability of the liposomes and the retention of fluorescence throughout the storage period.

**Figure S3** details the step-by-step fabrication of the PDMS microfluidic chip, from photolithographic mold preparation to device assembly via plasma bonding, ensuring reproducibility in device fabrication.

**Figure S4** depicts the complete experimental setup used for droplet generation and collection, including syringe pumps, the PDMS microfluidic device, and the optical monitoring system.

Together, these supplementary data provide insight into the fabrication and characterization of the thermoresponsive liposomes, supporting the robustness and reproducibility of the experimental methodology described in the main manuscript.

**Figure S1**. Extraction of oleic acid from double-emulsion droplets leading to solvent pocket formation. Schematic illustration and time-lapse bright-field and fluorescence images showing water-in-oil-in-water droplets with lipid-oleic acid shells dispersed in an ethanol-containing aqueous phase with 1% (w/v) Pluronic F-127. During extraction, solvent removal leads to thinning of the oil shell and formation of a transient solvent pocket, visible as a bright central region in the final stage. The lipid phase contained fluorescently labeled lipids (18:1 Liss Rhod PE) for visualization. Scale bars, 20 μm.

**Figure S2**. Bright-field and fluorescence microscopy images of liposomes after 7 and 14 days of storage in solution at room temperature. The images demonstrate the structural integrity and fluorescence retention of the liposomes over the storage period. Samples were imaged in a glass-bottom dish covered with a coverslip.

**Figure S3**. Step-by-step process for fabricating a PDMS microfluidic chip used in double emulsion droplet generation. The design is created using CAD software, and the mold is formed on a silicon wafer using SU-8 photolithography and patterned with a direct-write ML3 system. PDMS is cast on the mold, cured at 70 °C, peeled off, and punched to create inlets and outlets. The device is sealed by plasma bonding to a glass slide for final application.

**Figure S4**. Microfluidic setup for droplet generation and collection. Schematic setup showing the PDMS-based microfluidic device connected to syringe pumps for controlled infusion of the inner, middle, and outer phases. Droplet formation was monitored in real time using an inverted fluorescence microscope equipped with a high-speed camera. The generated droplets were collected in a vial containing extraction medium for subsequent solvent removal and membrane formation.

**Description of Additional Supplementary Files**

**File Name**: **Movie S1**.

**Description**: Bright-field video (20× objective) of the first flow-focusing junction showing formation of water-in-oil emulsions by combining the inner aqueous phase with a lipidic middle phase (DPPC dissolved in oleic acid) at a lipid-to-inner-phase flow-rate ratio of 1.25.

**File Name**: **Movie S2**.

**Description**: Bright-field video (20× objective) of the second flow-focusing junction showing shear of the water-in-oil droplets by the outer aqueous phase to generate water-in-oil-in-water double emulsions. Lipid-to-inner-phase flow-rate ratio = 1.25; double-emulsion production frequency ≈ 470 s^-^¹.

**File Name**: **Movie S3**.

**Description**: Bright-field video (20× objective) of the second flow-focusing junction showing shear of the water-in-oil droplets by the outer aqueous phase to generate water-in-oil-in-water double emulsions. Lipid-to-inner-phase flow-rate ratio = 2.0; double-emulsion production frequency ≈ 790 s^-^¹.

**File Name**: **Movie S4**.

**Description**: Confocal 3D reconstruction (fluorescently labeled lipids) confirming a well-defined spherical geometry and a distinct core-shell structure.

**File Name**: **Movie S5**.

**Description**: Fluorescence video (controlled temperature) showing the red fluorescent dye encapsulated in the liposome cores.

**File Name**: **Movie S6**.

**Description**: Bright-field video (4× objective) of both the first and second flow-focusing junctions, showing formation of water-in-oil and water-in-oil-in-water emulsions; the field of view includes the corona resistor (a serpentine channel connecting the two flow-focusing regions).

1. ^*^ Corresponding author: Ming Li, School of Mechanical and Manufacturing Engineering, University of New South Wales, Sydney, NSW, 2052, Australia. E-mail address: ming.li3@unsw.edu.au.
